# microRNA Expression during Trophectoderm Specification

**DOI:** 10.1371/journal.pone.0006143

**Published:** 2009-07-03

**Authors:** Srinivas R. Viswanathan, Craig H. Mermel, Jun Lu, Chi-Wei Lu, Todd R. Golub, George Q. Daley

**Affiliations:** 1 Department of Biological Chemistry and Molecular Pharmacology, Harvard Medical School; Harvard Stem Cell Institute, Boston, Massachusetts, United States of America; 2 Broad Institute of Massachusetts Institute of Technology (MIT) and Harvard, Cambridge, Massachusetts, United States of America; 3 Department of Obstetrics, Gynecology and Reproductive Science, UMDNJ- Robert Wood Johnson Med School Piscataway, Piscataway, New Jersey, United States of America; 4 Howard Hughes Medical Institute, Boston, Massachusetts, United States of America; 5 Stem Cell Transplantation Program, Division of Pediatric Hematology/Oncology, Children's Hospital Boston and Dana Farber Cancer Institute, Boston, Massachusetts, United States of America; 6 Division of Hematology, Brigham and Women's Hospital, Boston, Massachusetts, United States of America; 7 Manton Center for Orphan Disease Research, Boston, Massachusetts, United States of America; The University of Hong Kong, China

## Abstract

**Background:**

Segregation of the trophectoderm from the inner cell mass of the embryo represents the first cell-fate decision of mammalian development. Transcription factors essential for specifying trophectoderm have been identified, but the role of microRNAs (miRNAs) in modulating this fate-choice has been largely unexplored. We have compared miRNA expression in embryonic stem cell (ESC)-derived trophectoderm and in staged murine embryos to identify a set of candidate miRNAs likely to be involved in trophectoderm specification.

**Results:**

We profiled embryonic stem cells (ESCs) as they were induced to differentiate into trophectodermal cells by ectopic expression of HRas/Q61L. We also profiled murine embryos at progressive stages of preimplantation development (zygote, 2-cell, 4-cell, 8-cell, morula, and blastocyst), which includes the time window in which the trophectoderm is specified in vivo. Q61L/H

**Conclusions:**

We describe miRNA expression changes that occur during trophectoderm specification and validate that our in vitro system faithfully recapitulates trophectoderm specification in vivo. By comparing our in vitro and in vivo datasets, we have identified a minimal set of candidate miRNAs likely to play a role in trophectoderm specification. These miRNAs are predicted to regulate a host of development-associated target genes, and many of these miRNAs have previously reported roles in development and differentiation. Additionally, we highlight a number of miRNAs whose tight developmental regulation may reflect a functional role in other stages of embryogenesis. Our embryo profiling data may be useful to investigators studying trophectoderm specification and other stages of preimplantation development.

## Introduction

MicroRNAs (miRNAs) comprise a recently discovered class of ∼22 nucleotide regulatory RNAs with diverse roles in development, differentiation, and oncogenesis [1]–[3]. miRNAs bind to complementary sites within the 3′UTRs of cognate mRNAs, leading to translational repression or cleavage[4] of the targeted messages. Up to 30% of human genes are computationally predicted to be miRNA targets [5], suggesting a central role for miRNAs in the post-transcriptional regulation of gene expression.

Recent reports suggest that embryonic development is accompanied by dynamic changes in miRNA expression. In *C. elegans*, the *lin-4* [6]–[8] and *let-7* [7] miRNAs are critical for temporal control of larval development. Profiling studies in zebrafish have shown strong expression of miRNAs during and after segmentation; most of these miRNAs show exquisite tissue specificity [9]. Temporally regulated expression of groups of miRNAs occurs between days 5.5 and 11.5 of prenatal murine development [10]–[14]. Recent studies report miRNA expression patterns during preimplantation development, although significant differences exist between these studies in terms of methology applied and expression patterns observed [15], [16]. Other studies suggest a role for miRNAs in murine organogenesis [11], [12], [17]–[19].

MicroRNAs have also been identified as critical mediators of cellular differentiation events. Expression of miR-181 in Lin^−^ hematopoietic progenitor cells leads to an increase in lymphoid (B-lineage) differentiation [20]. The muscle specific miRNA miR-133 is critical for repression of the splicing factor nPTB during myoblast differentiation [21]. ES cells express relatively few miRNAs, but there is a global increase in miRNA expression upon differentiation to embryoid bodies [22]. A cluster of ES-specific miRNAs that is repressed upon embryoid body formation has also been described [23]. The notion that these miRNA expression changes are functionally important is supported by the observation that genetic inactivation of the miRNA biogenesis pathway almost abrogates the ability of ES cells to properly differentiate [24], [25]. Therefore, the upregulation of miRNAs upon differentiation is likely to be essential for the silencing of the ES cell self-renewal machinery and/or the establishment of lineage-specific transcriptional programmes.

Given the accumulating evidence for miRNAs as regulators of cell fate decisions, we sought to characterize miRNA expression during the first cell fate choice of mammalian development – the segregation of the inner cell mass (ICM) from the trophectoderm (TE). Until the eight-cell stage of mouse development, blastomeres are totipotent and retain the ability to contribute to all embryonic and extra-embryonic cell lineages [26], [27]. At the late eight-cell stage, blastomeres increase cell-cell contacts to form a compacted morula; in doing so, the blastomeres undergo an apical-basal polarization [28]. Subsequent asymmetric cell divisions produce two distinct cell populations: an outside population in contact with the environment and an inside population completely surrounded by the outside cells [29]. As development proceeds to the blastocyst stage, the outside cell population becomes increasingly epithelialized and gives rise to the TE, derivatives of which comprise the fetal contribution to the placenta. The inside cell population forms the ICM, which subsequently segregates into two populations: the epiblast (which gives rise to all the tissues of the embryo) and the primitive endoderm (which gives rise to the yolk sac) [27].

Lineage-specific transcription factors that serve as critical modulators of ICM/TE segregation have recently been identified. The ICM-specific transcription factor *Oct4* [30] is important for maintaining segregation of the ICM and TE; *Oct4*-null embryos form a functional TE, but lack epiblast and yolk sac [31]. *Nanog* is an epiblast-specific transcription factor that acts in concert with *Oct4* and may block differentiation into primitive endoderm [32]. *Cdx2* is a TE-specific transcription factor that is critical for specifying TE [33], [34]; *Cdx2-*null embryos undergo apoptosis within the TE and suffer from preimplantation lethality [34].

**Figure 1:**
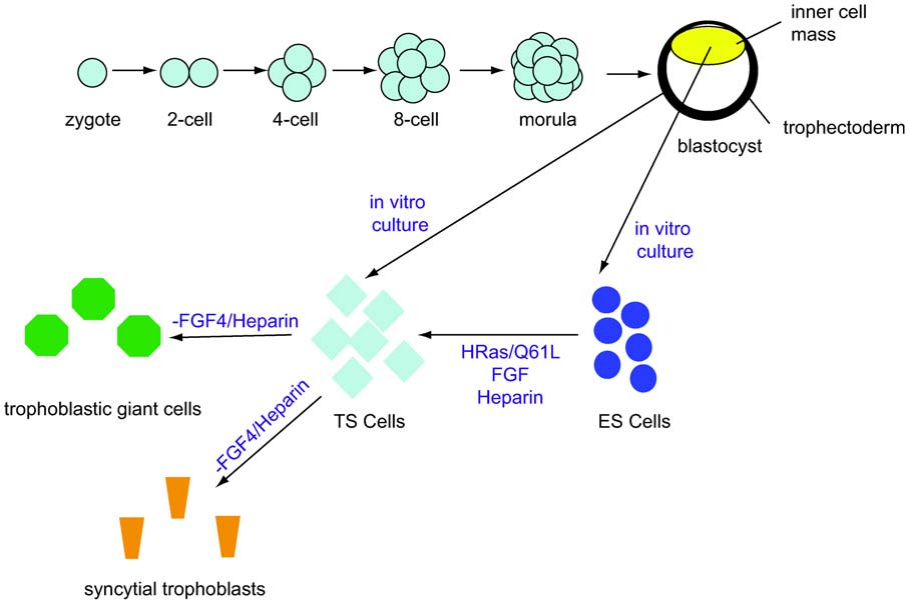
A schematic of trophectoderm specification *in vitro* and *in vivo*. In the developing mouse embryo, the trophectoderm is segregated from the inner cell mass between the morula and blastocyst stages of development. ESCs, derived from the inner cell mass, can be induced to differentiate into trophectodermal stem cells (ES-TS cells) by expression of HRas/Q61L and appropriate culture conditions. In the absence of FGF4, ES-TS and TS cells differentiate into trophectodermal cell types, such as trophoblastic giant cells and syncytial trophoblasts.

Cells from the ICM can be cultured and grown as ES cells, which differentiate into embryonic cell types *in vitro*. Trophectoderm-derived trophoblastic stem cells (TS cells) differentiate into extraembryonic placental cell types (Figure 1) [35]. While mouse ES cells cannot spontaneously differentiate into TE, we have recently shown that mouse ES cells can be homogenously driven toward TE fate by doxycycline-inducible expression of a constitutively active form of Ras (HRas/Q61L). Clonally isolated ES-derived TS cells (ES-TS cells) are phenotypically equivalent to blastocyst-derived TS cells (BD-TS cells) in *in vitro* and *in vivo* assays. Furthermore, Ras-MAPK signaling induces *Cdx2* expression, and Erk2 is apically localized in the 8-cell embryo; this suggests an early role for Ras-MAPK signaling in specification of TE *in vivo* and indicates that our *in vitro* differentiation system faithfully recapitulates embryonic TE specification [36].

In this study, we performed miRNA expression profiling on iRas-ES cells as they differentiated *in vitro* into TE. In parallel, we performed miRNA expression profiling on mouse embryos at various stages of preimplantation development. Our dataset reveals that miRNAs display dynamic changes in expression upon TE specification both *in vitro* and *in vivo*, and suggests a defined set of candidate miRNAs that may serve as regulators of the ICM/TE cell-fate choice.

## Results and Discussion

### miRNA Profiling During ES-TS Transition

**Figure 2:**
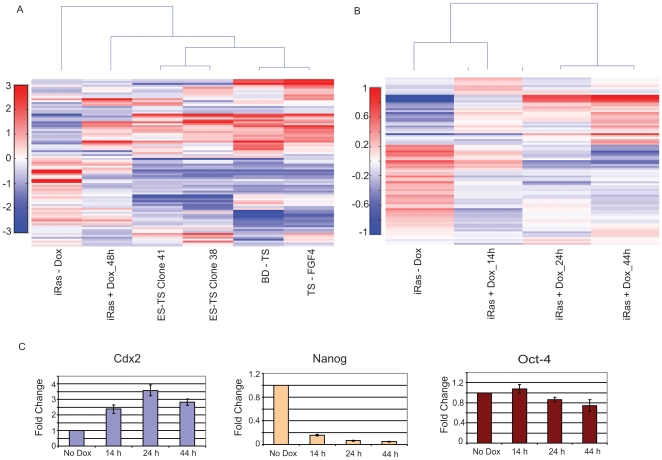
miRNA expression profiling during *in vitro* TE differentiation. a) unsupervised hierarchical clustering of miRNA expression to compare iRas-ESCs, iRas-ESC-derived TS cells (ES-TS cells), and BD-TS cells. b) unsupervised hierarchical clustering miRNA expression in iRas-ESCs after various time points of HRas/Q61L induction. c) quantitative PCR analysis of ES-specific (Oct-4 and Nanog) and TE-specific (Cdx2) marker genes after various time points of HRas/Q61L induction.

We initially sought to characterize miRNA expression during TE specification *in vitro*, and to determine the extent to which our ES*-*derived TS cells are molecularly similar to TS cells derived from the blastocyst. In our initial analysis, we compared miRNA expression profiles in the following samples: uninduced iRas-ESCs, iRas-ESCs in which HRas/Q61L expression had been induced with doxycycline for 48 hrs (iRas+Dox_48 hrs), two independent ES-derived TS clones (ES-TS Clone 38 and ES-TS Clone 41), blastocyst-derived TS cells (BD-TS), and BD-TS cells induced to differentiate into placental cell types by withdrawal of FGF4 (TS-FGF4). We observed dynamic changes in miRNA expression upon both ESC differentiation and BD-TS cell differentiation (Fig. 2a). We performed unsupervised hierarchical clustering to group samples based on their miRNA expression profiles, and observed that ES-TS Clone 38 and ES-TS Clone 41 clustered closely with BD-TS cells on the basis of their miRNA expression signatures. Therefore, our *in vitro* derived ES-TS cells closely resemble BD-TS cells on the basis of miRNA expression. Interestingly, the iRas+Dox_48 h sample clustered more closely with TS cells than with ES cells, indicating that many of the miRNA expression differences between TS cells and ES cells are established within two days of differentiation. Although ES-TS cells and BD-TS cells are highly similar in terms of overall miRNA expression and functional properties [36], we nonetheless identified 36 miRNAs that were differentially expressed more than 2-fold between these cell samples (Fig. S1 and Table S1). Together, these data support the notion that miRNA expression patterns are highly specific identifiers of developmental state [3].

To more closely analyze the kinetics of miRNA expression changes during TE specification, we performed miRNA expression profiling on iRas-ES cells after 14 h, 24 h, or 44 h of doxycycline treatment. Unsupervised hierarchical clustering revealed that uninduced iRas-ES cells (iRas-Dox) clustered with iRas-ES cells induced with doxycycline for 14 hrs (iRas+Dox_14 h), while iRas-ES_24 h and iRas-ES_44 h clustered together (Fig. 2b). We performed comparative marker selection analysis [37] to identify miRNAs that displayed statistically significant changes (cutoff of FDR<0.25) in expression during trophectodermal differentiation of ESCs. No miRNAs were significantly changed in expression level after 14 hrs of doxycycline treatment, despite the fact that Nanog was already strongly repressed and Cdx2 was strongly induced by this time point (Fig. 2c and data not shown). Thus, miRNAs may not be critical in the earliest stages of trophectoderm specification, but rather, may serve as developmental modulators that reinforce cell fate specification at later points in the differentiation process.

### Identification of Differentiation-Specific and Pluripotency-Specific miRNAs

#### miRNAs Associated with iRas-ESC Differentiation

**Figure 3:**
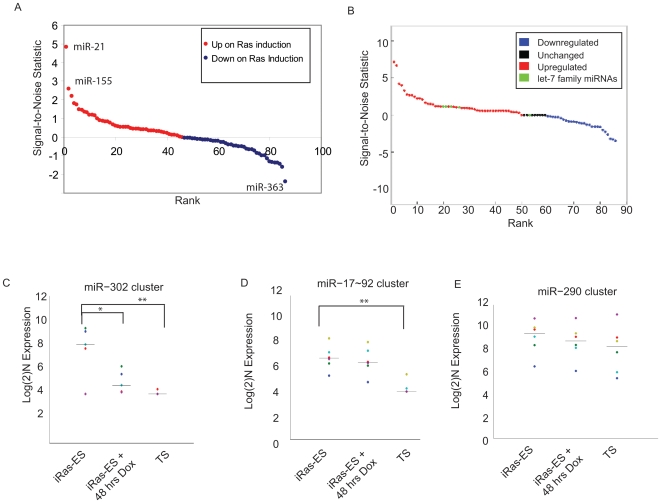
Identification of pluripotency-associated and differentiation-associated miRNAs. Comparative marker selection score plot for miRNAs differentially expressed between 0 and 44 hrs of HRas/Q61L induction (a) and for miRNAs showing expression difference between undifferentiated TS-cells and TS-cells induced to differentiate by withdrawal of FGF4 (b). Signal-to-noise statistic score is plotted vs. overall rank. For c–e, the expression of miRNAs within three pluripotency-associated miRNA clusters was assessed in: uninduced iRas-ESCs (iRas-ES), iRas-ESCs induced with doxycycline for 48 hrs (iRas-ES+48 hrs Dox), and BD-TS cells (TS). Individual family members within each cluster are demarked by different colors. c) miR-302 cluster (miRNA 302a, 302b, 302c, 302d, 367). *, p<0.05; **, p<0.01 by two-tailed unpaired Student's t-test. d) miRNA 17-92 cluster (miRNA 17, 18a, 19a, 20a, 19b–1, 92a–1). *, p<0.05; **, p<0.01 by two-tailed unpaired Student's t-test. e) miRNA 290 cluster (miRNA-290, 291a–3p, 292–3p, 293, 294, 295)

To determine whether any miRNAs showed significant changes in expression by 44 hrs of Hras/Q61L induction, we compared the iRas-Dox and iRas+Dox_44 h samples by comparative marker selection analysis. A total of 43 miRNAs were differentially expressed between the samples (Fig. 3a and Table S2, SNR Score >0.5 or <0.5 cutoff). Of these, 25 miRNAs were significantly induced upon trophectodermal differentiation, and 18 miRNAs were significantly downregulated. miRNAs induced upon differentiation may act to positively specify trophectoderm, or may serve to assist differentiation by silencing the self-renewal machinery. miRNAs downregulated upon differentiation may serve either as repressors of trophectoderm specification in the pluripotent state, or as active promoters of pluripotency.

#### miRNAs Associated with BD-TS Cell Differentiation

We also compared BD-TS cells with BD-TS cells induced to differentiate by withdrawal of FGF4. A number of miRNAs were changed in expression upon differentiation (Fig. 3b, Fig. S5, and Table S3). Notable among these were multiple members of the let-7 family of miRNAs, which were strongly induced upon BD-TS differentiation. let-7 miRNAs have been associated with the differentiated state in multiple other contexts [38], and consist of multiple family members located at several different genomic loci. Our observation that let-7 miRNAs are co-regulated upon BD-TS differentiation is consistent with recent reports that members of the let-7 family can be co-ordinately regulated in a post-transcriptional fashion [39]–[42]. Interestingly, we also noted that miR-24 was strongly induced upon BD-TS differentiation; miR-24 contains a perfect seed match to a site within the Cdx2 3′-UTR, and is computationally predicted to target Cdx2 by TargetScan, PicTar, and miRTIF [43]) (Table S3). Levels of Cdx2 decline as BD-TS cells differentiate [35], and our results suggest miR-24 may contribute to Cdx2 repression in this context.

#### miRNAs Associated with the Pluripotent State

Several ESC-specific miRNAs have been previously described, and we thus sought to characterize the expression patterns of these miRNAs in our system. We compared expression of miRNAs from three different ESC-associated miRNA clusters: (miR-302 cluster [44]; miR-290 cluster [23]; miR17-92 cluster[45]) in iRas-Dox, iRas+Dox_48 h, and BD-TS cells. Interestingly, we observed that each of the miRNA clusters we examined showed a slightly different expression pattern in these samples. Expression of the miR-302 cluster was highly ES-specific (Fig. 3c); levels of miR-302 miRNAs were downregulated upon HRas/Q61L induction, and further decreased in BD-TS cells. Expression of the miR-17-92 cluster was also higher in iRas-ESCs than in BD-TS cells, but only a modest decrease in expression occurred within 48 hrs of Hras/Q61L induction (Fig. 3d). Downregulation of the miR-17-92 cluster may thus be a late event in trophectodermal differentiation. Expression of the miR-290 cluster was comparable in iRas-ESCs, iRas-ES+Dox_48 hrs, and BD-TS cells (Fig. 3e). Consistent with this observation, the miR-290 cluster has been reported to be expressed in both ESCs and BD-TS cells [46]. Therefore, expression of this cluster may not be limited to ESCs, but rather, may be a more general feature of stem cell populations.

### miRNA Expression During Pre-Implantation Embryogenesis

To obtain a comprehensive portrait of miRNA expression during preimplantation development, we collected embryos at the zygote, 2-cell, 4-cell, 8-cell, morula, and blastocyst stages for profiling analysis. We began by analyzing expression of the miR-290, miR-302, and miR-17∼92 clusters in staged embryos. Although these clusters are all highly expressed in undifferentiated ESCs, and have been studied in the context of ESC differentiation *in vitro*, their temporal expression in the early embryo has not yet been characterized [47]. Therefore, it is unclear whether these miRNAs play a physiologically significant role in maintainance of the stem cell pool *in vivo*. We observed expression of the miR-290 cluster beginning at the 4-cell stage, and steadily increasing through the blastocyst stage. Our *in vitro* expression data (Fig. 3e) coupled with our embryo expression data suggest that the miR-290 cluster may play a critical role in early development and is likely to be expressed throughout the early embryo. Consistent with this notion, a genetic knockout of the miR-290 cluster is early embryonic lethal [47]. Expression of the miR-17∼92 cluster was variable throughout early development, with a modest increase between the morula and blastocyst stages of development. The miR-302 cluster was not detected at appreciable levels in the embryos profiled. Although this cluster may be expressed at levels too low to be detectable by our profiling method, our data suggest that it may be more significant in maintaining ESCs in culture than in regulating early development.

**Figure 4:**
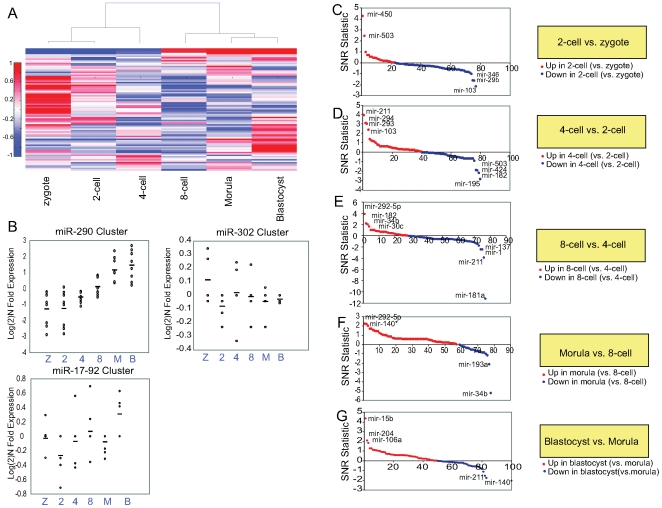
miRNA expression during preimplantation murine development. a) Unsupervised hierarchical clustering of miRNA expression for embryos at various developmental stages. b) row-normalized log(2) expression for ESC-specific miRNA clusters during embryonic development. Z, zygote; 2, 2-cell embryo; 4, 4-cell embryo; 8, 8-cell embryo; M, morula; B, blastocyst. c–g) comparative marker selection score plots for miRNAs differentially expressed between sequential stages of embryonic development. b) 2-cell vs. zygote. c) 4-cell vs. 2-cell. d) 8-cell vs. 4-cell. e) morula vs. 8-cell. f) blastocyst vs. morula. Signal-to-noise statistic is plotted against overall rank.

Further analysis of our embryo profiling data revealed a number of interesting features. First, unsupervised hierarchical clustering revealed that embryos clustered in developmental sequence (Fig. 4a), suggesting that incremental changes in miRNA expression occur at each stage of development. Second, pairwise comparative marker selection analysis revealed that only a small number of miRNAs are strongly induced or repressed at any given stage of development (Fig. 4c–g and Supplementary Data). Strikingly, several miRNAs showed dramatic directional changes in expression between successive stages of development, suggesting that they are strongly expressed only during narrow time windows. For example, miR-503 was among the most induced miRNAs between the zygote and 2-cell stages of development; between the 2-cell and 4-cell stages, it was among the most repressed (Fig. 4c–d). miR-34b showed a similar pattern between the 4-cell and morula stages (Fig. 4e–f), and miR-140* and miR-211 showed similar patterns between the 8-cell and blastocyst stages of development (Fig. 4f–g and Table S7). miR-182 was among the most downregulated miRNAs between the 2-cell and 4-cell stages of development; between the 4-cell and 8-cell stages, it was among the most induced (Fig d–e). miR-103 showed a similar pattern between the zygote and 4-cell stages (Fig. c–d). This suggests an exquisite temporal specificity to miRNA expression within the early embryo, and indicates that each embryonic stage is defined by a characteristic miRNA signature. Moreover, several miRNAs may serve as temporally regulated switches that tightly modulate developmental transitions. Third, there were very few changes in miRNA expression between the zygote and 2-cell stages of embryogenesis, consistent with the fact that zygotic genome activation occurs at the late 2-cell stage in the mouse.

### Candidate miRNAs Involved in Trophectoderm Specification

**Figure 5:**
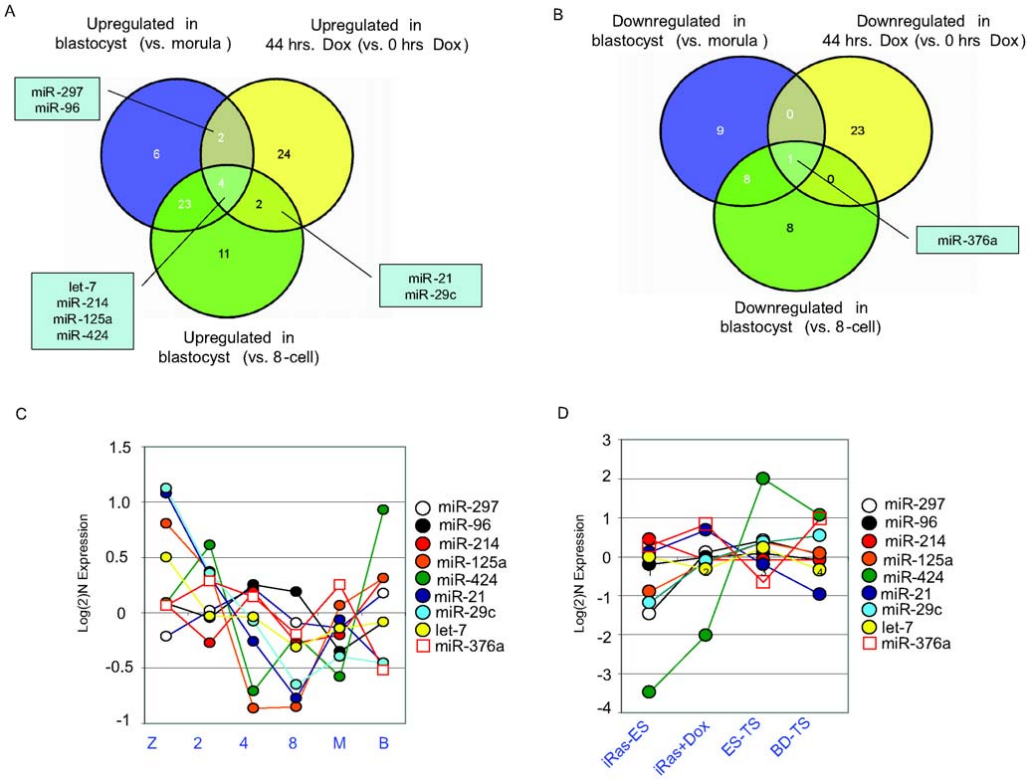
Candidate miRNAs involved in trophectoderm specification and maintenance of pluripotency. a) Venn diagram showing miRNAs that are differentially expressed during trophectoderm specification both *in vitro* and *in vivo*. b) Venn diagram showing candidate pluripotency-specific miRNAs that are expressed highly both in undifferentiated ESCs and in blastocysts. c) Expression of the nine candidate miRNAs established in Fig. 5a–b) during embryonic development. The average expression across all let-7 family members is depicted as a single circle. d) Expression of the nine candidate miRNAs established in Fig. 5a–b) in iRas-ESCs, iRas+Dox_48 hrs, ES-TS (signal averaged for Clone 38 and Clone 41), and BD-TS cells. The average expression across all let-7 family members is depicted as a single circle.

The trophectoderm is specified between the morula and blastocyst stages of embryonic development, although some of the cues that initiate specification may occur at the late 8-cell stage and other factors that bias fate choice may occur even earlier [48]. Our profiling data contains information about the changes in miRNA expression that occur when the trophectoderm is specified in the mammalian embryo, as well as when ESCs are differentiated into trophectodermal cells. Therefore, to identify candidate miRNAs with likely physiological significance, we compared our *in vitro* and *in vivo* datasets to identify overlapping miRNAs (Comparative Marker Selection score cut-off of +/− 0.5 used for these analyses) (Fig. 5). Comparative marker selection revealed 48 miRNAs that were upregulated between the morula and blastocyst stages or between the 8-cell embryo and blastocyst stages; this represents the time window of trophectoderm lineage specification in the embryo. 32 miRNAs were upregulated in iRas+Dox _44 h as compared to iRas-Dox ESCs. Eight miRNAs (“TE_up miRNAs”) were common to both datasets: miR-297, miR-96, miR-21, miR-29c, let-7, miR-214, miR-125a, and miR-424 (Fig. 5a). We performed an equivalent analysis to identify miRNAs that are downregulated upon trophectoderm specification both *in vitro* and *in vivo* and identified one candidate miRNA, miR-376a (Fig. 5b).

We investigated the expression of these nine candidate miRNAs (eight TE_up miRNAs in addition to miR-376a) in all preimplantation embryonic stages, ESCs, ES-TS cells, and BD-TS cells (Fig. 5c–d). The eight TE_up miRNAs were detectable in the zygote (and thus presumably maternally inherited), downregulated between the 2-cell through 8-cell stages, and induced from the the morula through blastocyst stages (Fig. 5c). miR-376a was induced between the 8-cell and morula stages, and rapidly downregulated upon blastocyst formation (Fig. 5c). Consistent with the *in vivo* data, levels of most of the TE_up miRNAs were increased upon HRas/Q61L induction, and were higher still in ES-TS cell clones (Fig. 5d). In general, levels of the TE_up miRNAs trended higher in BD-TS cells as compared with iRas ESCs, although the magnitude of expression change was not as dramatic as for ES-TS cells compared with iRas-Dox ESCs; this may be partially due to the fact that BD-TS cells are derived from a different mouse strain than the iRas-ESCs and have been derived under different culture conditions (Fig. 5d).

We predicted target genes for these nine candidate miRNAs using TargetScan software and analyzed gene pathways enriched in this list of predicted targets using the PANTHER gene ontogeny database. Several pathways were more highly enriched in this gene list as compared to a list of predicted targets of 9 random miRNAs (Table 1 and Table 2). The Ras and MAPK/p38 pathways were significantly enriched in the target gene list, consistent with the notion that RAS-MAPK signalling specifies trophectoderm both *in vitro* and *in vivo* [36]. Notably, the integrin signalling pathway was also significantly enriched in our target gene list, consistent with a central role for contact-mediated signalling in specification of the trophectoderm and implantation [49]–[51]. Cell-cell contacts likely play a key role in specifying outer cells of the compacted embryo as trophectoderm and inner cells of the embryo as the inner cell mass [48]. When miRNA targets were predicted using miRBase, the integrin signalling pathway was again enriched, although this prediction algorithm did not detect enrichment of RAS-MAPK signaling (Table S10). The high false positive rates associated with currently available target prediction algorithms are a significant caveat to this type of analysis. Nonetheless, these nine candidate miRNAs likely target hundreds of genes to regulate a complex differentiation program that specifies the trophectoderm.

**Table 1:** Predicted target genes (using TargetScan) for candidate miRNAs involved in trophectoderm specification were classified by gene ontology.

Pathway	# in Ref	# in List	expected	+/−	P value
Unclassified	26616	2058	2256.18	−	7.40E-29
Integrin signalling pathway	263	76	22.29	+	4.21E-17
EGF receptor signaling pathway	153	43	12.97	+	5.44E-09
Ras Pathway	95	32	8.05	+	2.32E-08
Wnt signaling pathway	408	77	34.59	+	4.24E-08
PDGF signaling pathway	186	46	15.77	+	6.70E-08
FGF signaling pathway	143	39	12.12	+	9.98E-08
PI3 kinase pathway	121	33	10.26	+	2.00E-06
p38 MAPK pathway	61	22	5.17	+	5.10E-06

Only pathways with p<1×10^−5^ are listed.

**Table 2:** Predicted target genes (using TargetScan) for 9 random miRNAs classified by gene pathway ontology.

Pathways	# in REF	# in List	Expected	+/−	P value
Unclassified	26616	1681	1870.07	−	4.56E-31
Wnt signaling pathway	408	104	28.67	+	7.34E-26
Cadherin signaling pathway	204	65	14.33	+	1.21E-20
PDGF signaling pathway	186	46	13.07	+	1.51E-10
Angiogenesis	258	51	18.13	+	2.54E-08
Endothelin signaling pathway	97	29	6.82	+	3.52E-08
EGF receptor signaling pathway	153	36	10.75	+	1.56E-07
FGF signaling pathway	143	34	10.05	+	3.49E-07
TGF-beta signaling pathway	154	34	10.82	+	2.08E-06

Only pathways with p<1×10−5 are shown.

Strikingly, we also noted that six of the nine miRNAs on our candidate list have previously defined roles in development and differentiation. miR-21 is upregulated upon differentiation of the leukaemia cell line HL-60 [52]; furthermore, in ESCs, the transcription factor REST maintains self-renewal and pluripotency through suppression of miR-21 [53]. The let-7 family of miRNAs is strongly associated with differentiation in multiple settings [38]. miR-214 has been implicated in specification of muscle cell types by modulation of Hedgehog signalling [54]. miR-125 is strongly induced upon neuronal differentiation of embryonal carcinoma cells, and may be involved in a developmental feedback loop during neural stem cell commitment [41], [55]. miR-424, which showed the most dramatic TE-specific upregulation, stimulates the monocyte/macrophage differentiation program [56]. miR-376a lies within a large imprinted cluster of miRNAs unique to placental mammals [57]. Given their association with developmental processes in other contexts, these miRNAs thus serve as attractive candidates for modulators of the ICM/TE fate choice.

miRNAs are emerging as key regulators of development and differentiation. Here, we present comprehensive miRNA profiling data for preimplantation embryonic development and, with the aid of an *in vitro* ES-differentiation system, identify a set of 9 candidate miRNAs with potential roles in trophectoderm specification. Functional characterization of large numbers of miRNAs in early embryos is unfeasible given the technical expertise and time commitment required. Our approach demonstrates that an ESC differentiation model can be used to substantially narrow the list of attractive candidates. Further functional characterization of these candidate miRNA as well as determination of their key target genes are exciting avenues for future investigation.

## Methods

### Cell Culture

iRas-ES cells were cultured as previously described [36]. Differentiation to trophectoderm was induced by culturing in the presence of 1 µg/ml doxycycline for the times indicated.

### Embryo Collection

CD1 female mice (Charles River) were superovulated by PMSG (7.5 IU) and HCG (7.5 IU). Embryos were collected at 1.5 dpc (1-cell stage), cumulus cells were removed by 0.03% Hyaluronidase, and embryos were allowed to develop *in vitro* in KSOM+AA (Speciality Media) to various developmental stages before being collected for miRNA profiling.

### RT-PCR

Total RNA was collected with Trizol and reverse transcribed using SuperScript III Reverse Transcriptase (Invitrogen). cDNA was amplified by quantitative PCR (annealing temperature 56°) using SYBR Green (Stratagene) as an indicator dye. Fold change was calculated using the ΔΔCt method with actin used as an internal control. Primer sequences are available upon request.

### miRNA Profiling

All samples were collected in Trizol and stored at −80°C until profiling. For embryo samples, each biological replicate consisted of at least 100 developmentally-staged embryos. Small RNA species were isolated and miRNAs were profiled using a bead-based detection system containing 435 miRNA probes using our previously described platform [3].

### Data Analysis

For each set of experiments, miRNA expression levels were normalized against the mean expression across all conditions for that experiment. Hierarchical clustering (Pearson correlation distance metric, complete linkage) was performed using the top 20% of miRNAs by variability. miRNAs that were differentially expressed between classes were ranked according to the Signal-to-Noise Ratio (SNR) test statistic, and significance for each miRNA was determined using the Comparative Marker Selection Suite using default settings [37].

For each sample, signal was averaged over replicates prior to clustering. Replicates clustered closely together as shown in Supplementary Data (Supplementary Figures S2, S3, S4). For embryo experiments, each biological replicate consisted of a pool of 100 staged embryos.

To identify candidate miRNAs involved in TE specification, we identified all miRNAs with SNR scores >0.5 or <0.5 from our *in vitro* and *in vivo* datasets, and checked for overlapping miRNAs between the sets. For this analysis, all let-7 family members were considered indistinguishable. For gene ontology analysis, we predicted target genes of all nine candidate miRNAs using TargetScan, and used this gene list as an input for the PANTHER gene pathway database. As a control, the total list of miRNAs profiled was randomized in order and 9 miRNAs were selected (miR-452, miR-7, miR-205, miR-15a, miR-144, miR-183, miR-463, miR-25, miR-99a), targets and pathway ontology was analyzed as for the candidate list.

## Supporting Information

Figure S1 Plot of miRNAs differentially expressed in ES-TS cells and BD-TS cells as determined by comparative marker selection. Red, higher expression in BD-TS cells than in ES-TS cells. Blue, higher expression in ES-TS cells than in BD-TS cells. miRNAs above and below dotted lines are differentially expressed more than 2-fold.
(0.04 MB DOC)

Figure S2 Clustering of replicate samples for heatmap shown in Fig. 2a.
(0.06 MB DOC)

Figure S3 Clustering of replicate samples for heatmap shown in Fig. 2c
(0.07 MB DOC)

Figure S4 Clustering of replicate samples for heatmap shown in Fig. 4a.
(0.06 MB DOC)

Figure S5 Differentially expressed miRNAs for TS cells induced to differentiate by withdrawal of FGF4. Values plotted as ranked fold change.
(0.04 MB DOC)

Table S1 Comparison of miRNA expression in BD-TS vs ES-TS cells by comparative marker selection. Data are sorted by SNR statistic score and only scores >0.5 or <−0.5 are shown.
(0.03 MB DOC)

Table S2 Comparative marker selection analysis on iRas-ES cells induced for 44 hrs vs. iRas-ES cells cultured without doxycycline. Data are sorted by SNR statistic score and only scores >0.5 or <−0.5 are shown.
(0.06 MB DOC)

Table S3 miRNAs differentially expressed upon TS differentiation. miRNAs with expression changes of SNR +/− 0.5 are shown.
(0.03 MB DOC)

Table S4 Comparative marker selection analysis on zygotes vs. 2-cell embryos. Only SNR >0.5 or <−0.5 are shown.
(0.05 MB DOC)

Table S5 Comparative marker selection analysis on 2-cell embryos vs. 4-cell embryos. Only SNR scores of >0.5 or <0.5 are shown.
(0.05 MB DOC)

Table S6 Comparative marker selection analysis on 4-cell embryos vs. 8-cell embryos. Only SNR scores of >0.5 or <0.5 are shown.
(0.07 MB DOC)

Table S7 Comparative marker selection analysis on 8-cell vs. morula. Only SNR scores of >0.5 or <0.5 are shown.
(0.07 MB DOC)

Table S8 Comparative marker selection analysis on morula vs. blastocyst. Only SNR scores of >0.5 or <0.5 are shown.
(0.06 MB DOC)

Table S9 Comparative marker selection analysis on 8-cell vs. blastocyst. Only SNR scores >0.5 or <−0.5 are shown.
(0.07 MB DOC)

Table S10 Predicted target genes using (miRBASE)for candidate miRNAs involved in trophectoderm specification were classified by gene ontology. Pathways with p<0.05 are shown.
(0.03 MB DOC)
