# Supplementary material for: microRNA Expression during Trophectoderm Specification
Source: PLoS One. 2009 Jul 3;4(7):e6143. doi: 10.1371/journal.pone.0006143 (PMC2702083; doi:10.1371/journal.pone.0006143)
Supplement: Figure S1 — Plot of miRNAs differentially expressed in ES-TS cells and BD-TS cells as determined by comparative marker selection. Red, higher expression in BD-TS cells than in ES-TS cells. Blue, higher expression in ES-TS cells than in BD-TS cells. miRNAs above and below dotted lines are differentially expressed more than 2-fold. (0.04 MB DOC) [file pone.0006143.s001.doc]

**
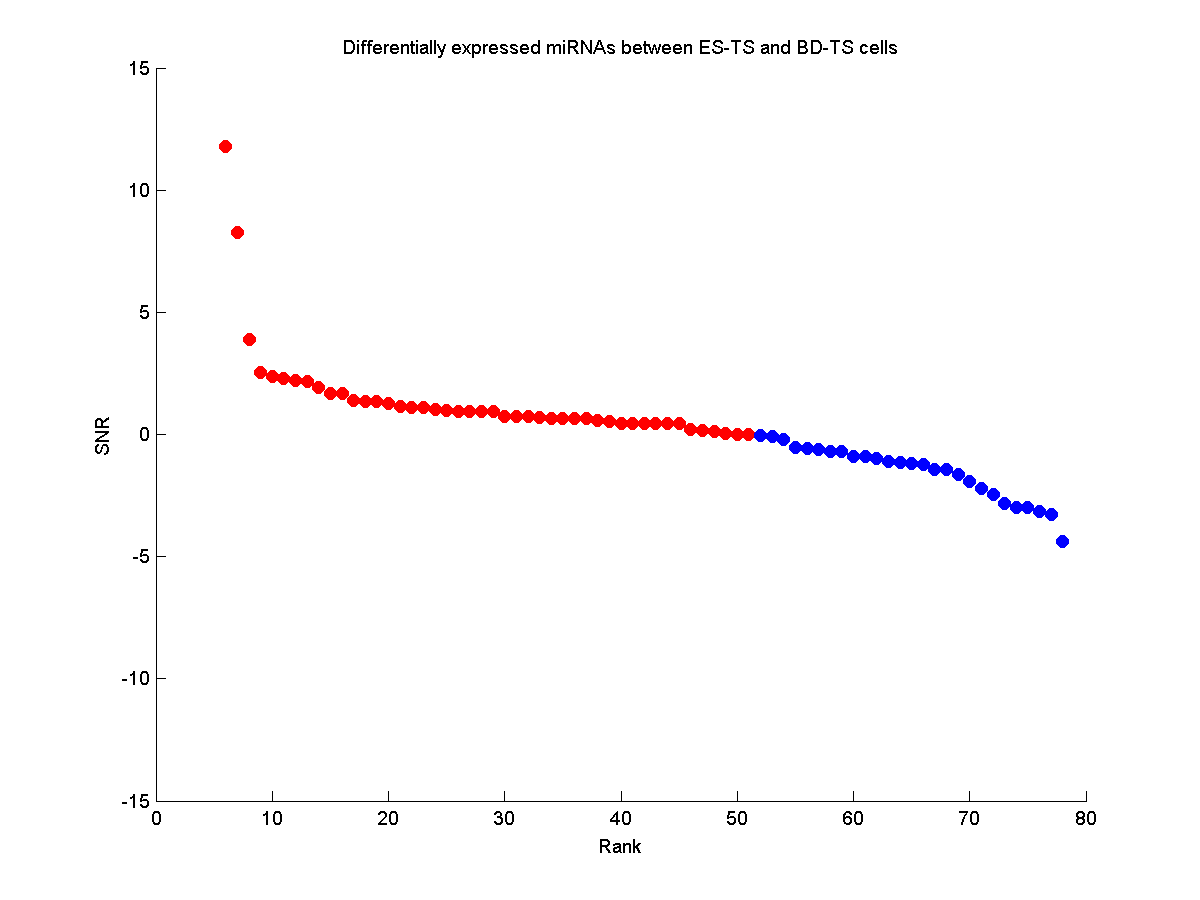
**

**Figure S1.** Plot of miRNAs differentially expressed in ES-TS cells and BD-TS cells as determined by comparative marker selection. Red, higher expression in BD-TS cells than in ES-TS cells. Blue, higher expression in ES-TS cells than in BD-TS cells. miRNAs above and below dotted lines are differentially expressed more than 2-fold.
