# Supplementary figures and images for: microRNA Expression during Trophectoderm Specification
Source: PLoS One. 2009 Jul 3;4(7):e6143. doi: 10.1371/journal.pone.0006143 (PMC2702083; doi:10.1371/journal.pone.0006143)

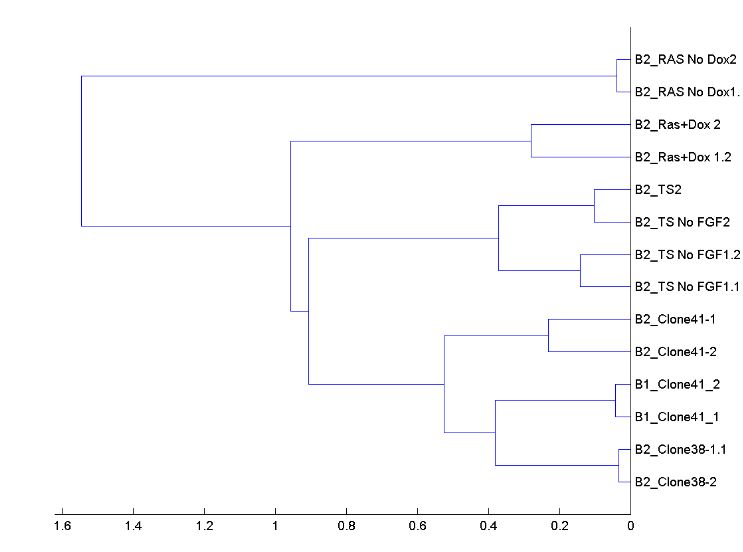


**Figure S2.** Clustering of replicate samples for heatmap shown in Fig. 2a.

Supplement: Figure S2 — Clustering of replicate samples for heatmap shown in Fig. 2a. (0.06 MB DOC) [file pone.0006143.s002.doc]

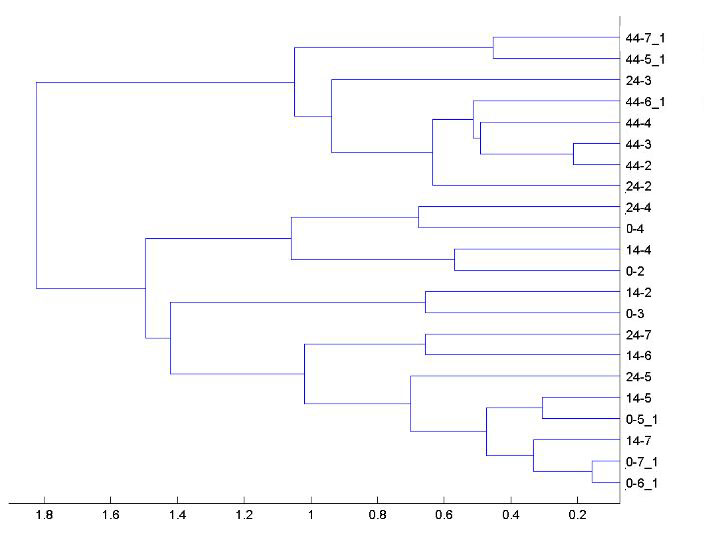


**Figure S3.** Clustering of replicate samples for heatmap shown in Fig. 2c

Supplement: Figure S3 — Clustering of replicate samples for heatmap shown in Fig. 2c (0.07 MB DOC) [file pone.0006143.s003.doc]

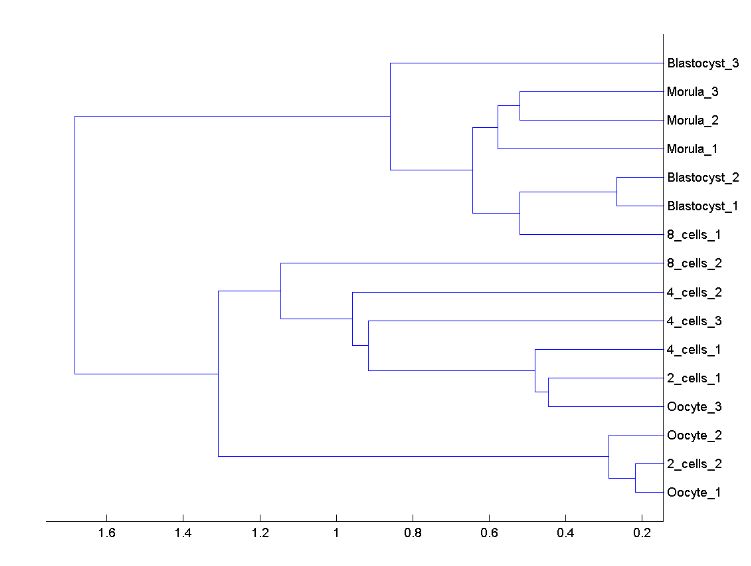


**Figure S4.** Clustering of replicate samples for heatmap shown in Fig. 4a.

Supplement: Figure S4 — Clustering of replicate samples for heatmap shown in Fig. 4a. (0.06 MB DOC) [file pone.0006143.s004.doc]
