# Supplementary material for: microRNA Expression during Trophectoderm Specification
Source: PLoS One. 2009 Jul 3;4(7):e6143. doi: 10.1371/journal.pone.0006143 (PMC2702083; doi:10.1371/journal.pone.0006143)
Supplement: Figure S5 — Differentially expressed miRNAs for TS cells induced to differentiate by withdrawal of FGF4. Values plotted as ranked fold change. (0.04 MB DOC) [file pone.0006143.s005.doc]

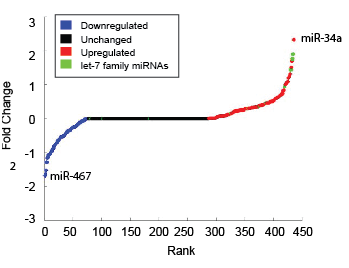


**Figure S5.** Differentially expressed miRNAs for TS cells induced to differentiate by withdrawal of FGF4. Values plotted as ranked fold change.
