# Supplementary material for: microRNA Expression during Trophectoderm Specification
Source: PLoS One. 2009 Jul 3;4(7):e6143. doi: 10.1371/journal.pone.0006143 (PMC2702083; doi:10.1371/journal.pone.0006143)
Supplement: Table S1 — Comparison of miRNA expression in BD-TS vs ES-TS cells by comparative marker selection. Data are sorted by SNR statistic score and only scores >0.5 or <−0.5 are shown. (0.03 MB DOC) [file pone.0006143.s006.doc]

**miRNA**  **SNR Score**
hmr-miR-34a_rfam7.0 11.806
hmr-miR-101_rfam7.0 8.262
hm-miR-189_rfam7.0 3.902
hmr-miR-25_rfam7.0 2.534
hmr-miR-103_rfam7.0 2.37
hmr-miR-212_rfam7.0 2.29
hmr-miR-205_rfam7.0 2.222
hmr-miR-7_rfam7.0 2.161
hmr-miR-199a_rfam7.0 1.909
hmr-miR-29c_rfam7.0 1.692
mr-miR-217_rfam7.0 1.665
hmr-miR-181a_rfam7.0 1.374
hmr-miR-132_rfam7.0 1.355
hmr-miR-136_rfam7.0 1.334
hmr-miR-10a_rfam7.0 1.282
hmr-miR-26b_rfam7.0 1.16
h-miR-224_rfam7.0 1.092
hmr-miR-99a_rfam7.0 1.085
hmr-miR-181c_rfam7.0 1.038
hmr-miR-29b_rfam7.0 0.968
hmr-miR-210_rfam7.0 0.951
hmr-miR-106b_rfam7.0 0.927
hmr-miR-203_rfam7.0 0.927
hmr-let-7b_rfam7.0 0.924
hmr-miR-208_rfam7.0 0.733
h-miR-217_rfam7.0 0.729
hmr-miR-142-5p_rfam7.0 0.722
hmr-miR-127_rfam7.0 0.679
hmr-let-7d_rfam7.0 0.668
h-miR-215_rfam7.0 0.652
hmr-miR-125a_rfam7.0 0.65
h-miR-199b_rfam7.0 0.641
hmr-miR-17-5p_rfam7.0 0.579
h-miR-105_rfam7.0 0.516
hmr-miR-92_rfam7.0 -0.524
hmr-miR-125b_rfam7.0 -0.555
hmr-miR-30b_rfam7.0 -0.625
hmr-miR-181b_rfam7.0 -0.7
hmr-miR-122a_rfam7.0 -0.715
m-miR-155_rfam7.0 -0.891
hmr-miR-146a_rfam7.0 -0.908
h-miR-211_rfam7.0 -0.98
m-miR-199b_rfam7.0 -1.096
mr-miR-211_rfam7.0 -1.142
hmr-miR-206_rfam7.0 -1.193
hmr-let-7i_rfam7.0 -1.22
hm-miR-15a_rfam7.0 -1.431
hmr-miR-15b_rfam7.0 -1.436
hmr-miR-204_rfam7.0 -1.619
hmr-let-7f_rfam7.0 -1.929
mr-miR-101b_rfam7.0 -2.212
hmr-let-7a_rfam7.0 -2.445
hmr-miR-19a_rfam7.0 -2.819
hmr-miR-200c_rfam7.0 -2.983
hmr-let-7c_rfam7.0 -2.997
h-miR-10b_rfam7.0 -3.166
hmr-miR-218_rfam7.0 -3.256
hmr-miR-126*_rfam7.0 -4.388

|  |  |
| --- | --- |
|  |  |

**Table S1.** Comparison of miRNA expression in BD-TS vs ES-TS cells by comparative marker selection. Data are sorted by SNR statistic score and only scores >0.5 or <-0.5 are shown.
