# Supplementary material for: microRNA Expression during Trophectoderm Specification
Source: PLoS One. 2009 Jul 3;4(7):e6143. doi: 10.1371/journal.pone.0006143 (PMC2702083; doi:10.1371/journal.pone.0006143)
Supplement: Table S2 — Comparative marker selection analysis on iRas-ES cells induced for 44 hrs vs. iRas-ES cells cultured without doxycycline. Data are sorted by SNR statistic score and only scores >0.5 or <−0.5 are shown. (0.06 MB DOC) [file pone.0006143.s007.doc]

| **Feature** | **Score** | **Feature P** | **FDR(BH)** |
| --- | --- | --- | --- |
| hmr-miR-21_rfam7.0 | 4.831685 | 0.001996 | 0.009647 |
| m-miR-155_rfam7.0 | 2.590087 | 0.001996 | 0.009647 |
| m-miR-467_rfam7.0 | 2.187831 | 0.001996 | 0.009647 |
| h-miR-155_rfam7.0 | 1.802827 | 0.01996 | 0.056017 |
| hmr-miR-28_rfam7.0 | 1.739698 | 0.001996 | 0.009647 |
| m-miR-466_rfam7.0 | 1.480283 | 0.001996 | 0.009647 |
| hsa-mir-24* | 1.454601 | 0.001996 | 0.009647 |
| hmr-miR-192_rfam7.0 | 1.34624 | 0.00998 | 0.034731 |
| hmr-miR-200c_rfam7.0 | 1.307204 | 0.001996 | 0.009647 |
| hmr-miR-222_rfam7.0 | 1.199243 | 0.00998 | 0.034731 |
| hmr-miR-29c_rfam7.0 | 1.182705 | 0.005988 | 0.024808 |
| hmr-miR-320_rfam7.0 | 1.149283 | 0.007984 | 0.0302 |
| hmr-miR-221_rfam7.0 | 1.00237 | 0.013972 | 0.041916 |
| hmr-miR-365_rfam7.0 | 0.911747 | 0.013972 | 0.041916 |
| hmr-miR-29a_rfam7.0 | 0.875558 | 0.017964 | 0.052096 |
| hmr-miR-125a_rfam7.0 | 0.84772 | 0.005988 | 0.024808 |
| hmr-miR-22_rfam7.0 | 0.836285 | 0.007984 | 0.0302 |
| hmr-miR-96_rfam7.0 | 0.7813 | 0.033932 | 0.089457 |
| hmr-miR-424_rfam7.0 | 0.686353 | 0.05988 | 0.140799 |
| hmr-miR-29b_rfam7.0 | 0.607275 | 0.06986 | 0.159943 |
| hmr-let-7e_rfam7.0 | 0.57782 | 0.07984 | 0.178105 |
| hsa-mir-425* | 0.556275 | 0.085828 | 0.186677 |
| hm-miR-199a*_rfam7.0 | 0.549817 | 0.101796 | 0.210864 |
| hmr-miR-135b_rfam7.0 | 0.539457 | 0.113772 | 0.224959 |
| hmr-miR-214_rfam7.0 | 0.538324 | 0.095808 | 0.203301 |
| hmr-miR-335_rfam7.0 | -0.5021 | 0.011976 | 0.038589 |
| hmr-miR-107_rfam7.0 | -0.52926 | 0.107784 | 0.218075 |
| h-miR-369-3p_rfam7.0 | -0.62831 | 0.0499 | 0.124038 |
| m-miR-376a_rfam7.0 | -0.67022 | 0.02994 | 0.0814 |
| hsa-miR-495 (j-mir-20) | -0.6734 | 0.057884 | 0.139887 |
| hm-miR-409-3p_rfam7.0 | -0.73899 | 0.035928 | 0.091934 |
| h-miR-302a*_rfam7.0 | -0.8661 | 0.001996 | 0.009647 |
| h-miR-367_rfam7.0 | -0.89484 | 0.001996 | 0.009647 |
| h-miR-494_rfam7.0 | -0.97869 | 0.001996 | 0.009647 |
| m-miR-376b_rfam7.0 | -0.99441 | 0.001996 | 0.009647 |
| hmr-miR-150_rfam7.0 | -1.13886 | 0.011976 | 0.038589 |
| h-miR-302c_rfam7.0 | -1.32273 | 0.001996 | 0.009647 |
| hmr-miR-130b_rfam7.0 | -1.33788 | 0.005988 | 0.024808 |
| hm-miR-410_rfam7.0 | -1.37933 | 0.001996 | 0.009647 |
| hm-miR-302a_rfam7.0 | -1.38085 | 0.001996 | 0.009647 |
| h-miR-302d_rfam7.0 | -1.45266 | 0.001996 | 0.009647 |
| h-miR-302b_rfam7.0 | -1.59974 | 0.001996 | 0.009647 |
| hm-miR-363 (j-mir-49) | -2.36969 | 0.001996 | 0.009647 |

**Table S2.** Comparative marker selection analysis on iRas-ES cells induced for 44 hrs vs. iRas-ES cells cultured without doxycycline. Data are sorted by SNR statistic score and only scores >0.5 or <-0.5 are shown.
