# Supplementary material for: microRNA Expression during Trophectoderm Specification
Source: PLoS One. 2009 Jul 3;4(7):e6143. doi: 10.1371/journal.pone.0006143 (PMC2702083; doi:10.1371/journal.pone.0006143)
Supplement: Table S3 — miRNAs differentially expressed upon TS differentiation. miRNAs with expression changes of SNR +/− 0.5 are shown. (0.03 MB DOC) [file pone.0006143.s008.doc]

miR SNR Score

hmr-miR-136_rfam7.0 7.172

hmr-miR-21_rfam7.0 6.663

hmr-miR-25_rfam7.0 4.211

hmr-miR-34a_rfam7.0 3.990

hmr-miR-23a_rfam7.0 3.313

hmr-miR-29c_rfam7.0 2.761

hmr-miR-125a_rfam7.0 2.658

hmr-miR-23b_rfam7.0 2.649

hmr-miR-22_rfam7.0 2.458

hmr-miR-199a_rfam7.0 2.235

hmr-miR-27b_rfam7.0 2.234

hmr-miR-29a_rfam7.0 2.106

hmr-miR-143_rfam7.0 1.689

hmr-miR-19b_rfam7.0 1.513

hmr-miR-27a_rfam7.0 1.467

hmr-miR-24_rfam7.0 1.444

m-miR-295_rfam7.0 1.191

hmr-miR-145_rfam7.0 1.178

hmr-miR-16_rfam7.0 1.155

hmr-let-7b_rfam7.0 1.151

hmr-let-7c_rfam7.0 1.150

hmr-miR-26a_rfam7.0 1.125

hmr-let-7a_rfam7.0 1.124

hmr-miR-30b_rfam7.0 1.102

hmr-miR-130a_rfam7.0 1.060

hmr-let-7d_rfam7.0 1.033

hmr-miR-193a_rfam7.0 0.975

m-miR-293_rfam7.0 0.961

hmr-miR-20a_rfam7.0 0.951

hmr-miR-424_rfam7.0 0.925

hmr-miR-99b_rfam7.0 0.847

hm-miR-182_rfam7.0 0.784

hmr-miR-26b_rfam7.0 0.633

hmr-miR-18a_rfam7.0 0.593

h-miR-302d_rfam7.0 0.577

hmr-miR-195_rfam7.0 0.577

hmr-miR-19a_rfam7.0 0.577

hmr-miR-210_rfam7.0 0.577

mr-miR-7b_rfam7.0 0.577

hmr-miR-135b_rfam7.0 0.577

m-miR-376b_rfam7.0 0.577

hm-miR-15a_rfam7.0 0.577

h-miR-106a_rfam7.0 0.577

hmr-miR-134_rfam7.0 0.577

hmr-miR-96_rfam7.0 0.553

hmr-miR-93_rfam7.0 -0.599

mr-miR-345_rfam7.0 -0.758

m-miR-297_rfam7.0 -0.854

hmr-miR-127_rfam7.0 -0.915

j-mir-41 (match mir-527*)-0.925

m-miR-434-5p_rfam7.0 -0.934

h-miR-369-3p_rfam7.0 -1.033

mr-miR-322_rfam7.0 -1.140

hmr-miR-15b_rfam7.0 -1.165

mr-miR-351_rfam7.0 -1.266

h-miR-503_rfam7.0 -1.327

m-miR-376a_rfam7.0 -1.555

h-miR-18b_rfam7.0 -1.567

mr-miR-291-5p_rfam7.0 -1.571

hmr-miR-33_rfam7.0 -1.608

hmr-miR-324-3p_rfam7.0 -2.025

m-miR-466_rfam7.0 -2.239

hsa-miR-503 (j-mir-51) -2.644

j-mir-26 -3.209

mr-miR-298_rfam7.0 -3.284

m-miR-467_rfam7.0 -3.464

**Table S3.** miRNAs differentially expressed upon TS differentiation. miRNAs with expression changes of SNR +/- 0.5 are shown.
