# Supplementary material for: microRNA Expression during Trophectoderm Specification
Source: PLoS One. 2009 Jul 3;4(7):e6143. doi: 10.1371/journal.pone.0006143 (PMC2702083; doi:10.1371/journal.pone.0006143)
Supplement: Table S4 — Comparative marker selection analysis on zygotes vs. 2-cell embryos. Only SNR >0.5 or <−0.5 are shown. (0.05 MB DOC) [file pone.0006143.s009.doc]

| **Feature** | **Score** | **Feature P** | **FDR(BH)** |
| --- | --- | --- | --- |
| hmr-miR-103_rfam7.0 | 2.155357 | 0.237525 | 0.831337 |
| m-miR-346_rfam7.0 | 1.642627 | 0.237525 | 0.831337 |
| hmr-miR-29b_rfam7.0 | 1.612832 | 0.237525 | 0.831337 |
| hmr-miR-106b_rfam7.0 | 1.035417 | 0.566866 | 0.890357 |
| h-miR-10b_rfam7.0 | 0.911736 | 0.61477 | 0.890357 |
| hmr-miR-204_rfam7.0 | 0.842418 | 0.566866 | 0.890357 |
| hm-let-7g_rfam7.0 | 0.84241 | 0.237525 | 0.831337 |
| hmr-miR-195_rfam7.0 | 0.807806 | 0.407186 | 0.890357 |
| mr-miR-292-3p_rfam7.0 | 0.723602 | 0.237525 | 0.831337 |
| hm-miR-1_rfam7.0 | 0.688752 | 0.237525 | 0.831337 |
| hmr-miR-338_rfam7.0 | 0.686753 | 0.654691 | 0.932102 |
| hmr-miR-30c_rfam7.0 | 0.644233 | 0.433134 | 0.890357 |
| hmr-miR-199a_rfam7.0 | 0.63865 | 0.61477 | 0.890357 |
| hmr-miR-181a_rfam7.0 | 0.626582 | 0.397206 | 0.890357 |
| hmr-miR-150_rfam7.0 | 0.579445 | 0.433134 | 0.890357 |
| hmr-miR-18a_rfam7.0 | 0.57735 | 0.862275 | 0.982036 |
| mr-miR-211_rfam7.0 | 0.57735 | 0.784431 | 0.982036 |
| h-miR-302c_rfam7.0 | 0.57735 | 0.784431 | 0.982036 |
| hmr-miR-214_rfam7.0 | 0.57735 | 0.862275 | 0.982036 |
| m-miR-294_rfam7.0 | 0.57735 | 0.756487 | 0.982036 |
| hmr-let-7f_rfam7.0 | 0.57735 | 0.784431 | 0.982036 |
| hmr-miR-17-5p_rfam7.0 | 0.529324 | 0.433134 | 0.890357 |
| hmr-miR-145_rfam7.0 | 0.520555 | 0.61477 | 0.890357 |
| m-miR-293_rfam7.0 | -0.54998 | 0.197605 | 0.831337 |
| hm-miR-182_rfam7.0 | -0.55347 | 0.161677 | 0.831337 |
| mr-miR-290_rfam7.0 | -0.70711 | 0.001996 | 0.013972 |
| mr-miR-351_rfam7.0 | -0.70711 | 0.001996 | 0.013972 |
| hmr-miR-323_rfam7.0 | -0.974 | 0.001996 | 0.013972 |
| hsa-miR-503 (j-mir-51) | -2.43402 | 0.001996 | 0.013972 |
| hmr-miR-450_rfam7.0 | -4.27021 | 0.001996 | 0.013972 |

**Table S4.**  Comparative marker selection analysis on zygotes vs. 2-cell embryos. Only SNR > 0.5 or <-0.5 are shown.
