# Supplementary material for: microRNA Expression during Trophectoderm Specification
Source: PLoS One. 2009 Jul 3;4(7):e6143. doi: 10.1371/journal.pone.0006143 (PMC2702083; doi:10.1371/journal.pone.0006143)
Supplement: Table S5 — Comparative marker selection analysis on 2-cell embryos vs. 4-cell embryos. Only SNR scores of >0.5 or <0.5 are shown. (0.05 MB DOC) [file pone.0006143.s010.doc]

| Feature | Score | Feature P | FDR(BH) |
| --- | --- | --- | --- |
| hmr-miR-195_rfam7.0 | 2.871635 | 0.197605 | 0.634731 |
| hsa-miR-503 (j-mir-51) | 2.245175 | 0.197605 | 0.634731 |
| hmr-miR-424_rfam7.0 | 1.981597 | 0.197605 | 0.634731 |
| hm-miR-182_rfam7.0 | 1.928159 | 0.197605 | 0.634731 |
| hmr-miR-450_rfam7.0 | 0.928616 | 0.407186 | 0.938016 |
| hmr-let-7a_rfam7.0 | 0.707107 | 0.826347 | 0.938016 |
| hmr-miR-24_rfam7.0 | 0.707107 | 0.826347 | 0.938016 |
| hmr-miR-141_rfam7.0 | 0.707107 | 0.826347 | 0.938016 |
| mr-miR-351_rfam7.0 | 0.707107 | 0.826347 | 0.938016 |
| hmr-miR-27b_rfam7.0 | 0.707107 | 0.826347 | 0.938016 |
| mr-miR-290_rfam7.0 | 0.707107 | 0.780439 | 0.938016 |
| hm-miR-149_rfam7.0 | 0.692984 | 0.826347 | 0.938016 |
| hmr-miR-30d_rfam7.0 | 0.64467 | 0.826347 | 0.938016 |
| mr-miR-34b_rfam7.0 | 0.626644 | 0.826347 | 0.938016 |
| hmr-miR-34c_rfam7.0 | 0.603451 | 0.39521 | 0.938016 |
| hmr-miR-125a_rfam7.0 | 0.567578 | 0.39521 | 0.938016 |
| hmr-miR-449_rfam7.0 | 0.539172 | 0.568862 | 0.938016 |
| hmr-miR-99a_rfam7.0 | 0.516354 | 0.826347 | 0.938016 |
| hmr-miR-107_rfam7.0 | -0.50154 | 0.187625 | 0.634731 |
| hmr-miR-324-5p_rfam7.0 | -0.52228 | 0.211577 | 0.634731 |
| hmr-miR-34a_rfam7.0 | -0.57735 | 0.001996 | 0.008383 |
| mr-miR-291-3p_rfam7.0 | -0.57735 | 0.001996 | 0.008383 |
| h-miR-302c_rfam7.0 | -0.57735 | 0.001996 | 0.008383 |
| hmr-miR-214_rfam7.0 | -0.57735 | 0.001996 | 0.008383 |
| hmr-let-7f_rfam7.0 | -0.57735 | 0.001996 | 0.008383 |
| hmr-miR-106b_rfam7.0 | -0.589 | 0.001996 | 0.008383 |
| hmr-miR-196a_rfam7.0 | -0.73441 | 0.001996 | 0.008383 |
| m-miR-346_rfam7.0 | -0.7926 | 0.237525 | 0.688003 |
| hmr-miR-18a_rfam7.0 | -0.96671 | 0.001996 | 0.008383 |
| hmr-miR-181a_rfam7.0 | -1.09284 | 0.001996 | 0.008383 |
| hmr-miR-338_rfam7.0 | -1.15451 | 0.001996 | 0.008383 |
| mr-miR-291-5p_rfam7.0 | -1.22084 | 0.001996 | 0.008383 |
| mr-miR-292-3p_rfam7.0 | -1.34391 | 0.001996 | 0.008383 |
| hmr-miR-103_rfam7.0 | -2.34963 | 0.001996 | 0.008383 |
| m-miR-293_rfam7.0 | -3.0058 | 0.001996 | 0.008383 |
| m-miR-294_rfam7.0 | -3.06733 | 0.001996 | 0.008383 |
| mr-miR-211_rfam7.0 | -3.90974 | 0.001996 | 0.008383 |

**Table S5.**  Comparative marker selection analysis on 2-cell embryos vs. 4-cell embryos. Only SNR scores of > 0.5 or <0.5 are shown.
