# Supplementary material for: microRNA Expression during Trophectoderm Specification
Source: PLoS One. 2009 Jul 3;4(7):e6143. doi: 10.1371/journal.pone.0006143 (PMC2702083; doi:10.1371/journal.pone.0006143)
Supplement: Table S6 — Comparative marker selection analysis on 4-cell embryos vs. 8-cell embryos. Only SNR scores of >0.5 or <0.5 are shown. (0.07 MB DOC) [file pone.0006143.s011.doc]

| Feature | Score | Feature P | FDR(BH) |
| --- | --- | --- | --- |
| hmr-miR-181a_rfam7.0 | 11.21672 | 0.237525 | 0.604609 |
| mr-miR-211_rfam7.0 | 3.90974 | 0.237525 | 0.604609 |
| hmr-miR-137_rfam7.0 | 2.419229 | 0.237525 | 0.604609 |
| hm-miR-1_rfam7.0 | 2.416939 | 0.237525 | 0.604609 |
| hmr-miR-193a_rfam7.0 | 1.777347 | 0.237525 | 0.604609 |
| hmr-let-7c_rfam7.0 | 1.45429 | 0.237525 | 0.604609 |
| hmr-miR-30a-5p_rfam7.0 | 1.311476 | 0.237525 | 0.604609 |
| hmr-miR-29c_rfam7.0 | 1.139726 | 0.566866 | 0.898351 |
| hmr-miR-23b_rfam7.0 | 1.113097 | 0.61477 | 0.898351 |
| hmr-miR-29b_rfam7.0 | 1.037317 | 0.61477 | 0.898351 |
| hmr-miR-143_rfam7.0 | 1.01547 | 0.566866 | 0.898351 |
| hmr-miR-324-5p_rfam7.0 | 0.968506 | 0.237525 | 0.604609 |
| hmr-miR-34c_rfam7.0 | 0.931687 | 0.654691 | 0.898351 |
| hmr-miR-18a_rfam7.0 | 0.873764 | 0.654691 | 0.898351 |
| hmr-miR-323_rfam7.0 | 0.816839 | 0.397206 | 0.842315 |
| hmr-let-7b_rfam7.0 | 0.781002 | 0.566866 | 0.898351 |
| hmr-miR-342_rfam7.0 | 0.748388 | 0.421158 | 0.842315 |
| hmr-miR-21_rfam7.0 | 0.736019 | 0.566866 | 0.898351 |
| hmr-miR-196a_rfam7.0 | 0.734413 | 0.654691 | 0.898351 |
| hmr-miR-199a_rfam7.0 | 0.69383 | 0.566866 | 0.898351 |
| hmr-miR-142-3p_rfam7.0 | 0.667928 | 0.654691 | 0.898351 |
| hmr-miR-33_rfam7.0 | 0.621111 | 0.666667 | 0.898351 |
| hm-let-7g_rfam7.0 | 0.617019 | 0.407186 | 0.842315 |
| hmr-miR-449_rfam7.0 | 0.598614 | 0.433134 | 0.846122 |
| hmr-miR-106b_rfam7.0 | 0.588996 | 0.654691 | 0.898351 |
| hmr-miR-23a_rfam7.0 | 0.57735 | 0.756487 | 0.898351 |
| hmr-miR-195_rfam7.0 | 0.57735 | 0.862275 | 0.898351 |
| hmr-miR-27a_rfam7.0 | 0.57735 | 0.862275 | 0.898351 |
| hmr-miR-214_rfam7.0 | 0.57735 | 0.784431 | 0.898351 |
| hmr-miR-145_rfam7.0 | 0.57735 | 0.862275 | 0.898351 |
| h-miR-302c_rfam7.0 | 0.57735 | 0.784431 | 0.898351 |
| hsa-miR-503 (j-mir-51) | 0.57735 | 0.756487 | 0.898351 |
| hmr-miR-34a_rfam7.0 | 0.57735 | 0.862275 | 0.898351 |
| hmr-miR-338_rfam7.0 | 0.556803 | 0.61477 | 0.898351 |
| hmr-miR-125b_rfam7.0 | 0.532612 | 0.566866 | 0.898351 |
| m-miR-106a_rfam7.0 | -0.60624 | 0.331337 | 0.818598 |
| m-miR-293_rfam7.0 | -0.67953 | 0.235529 | 0.604609 |
| hm-miR-149_rfam7.0 | -0.68655 | 0.185629 | 0.604609 |
| hmr-let-7a_rfam7.0 | -0.70711 | 0.001996 | 0.009863 |
| hmr-miR-424_rfam7.0 | -0.70711 | 0.001996 | 0.009863 |
| hmr-miR-30d_rfam7.0 | -0.78488 | 0.001996 | 0.009863 |
| hmr-miR-92_rfam7.0 | -0.79786 | 0.183633 | 0.604609 |
| hmr-miR-103_rfam7.0 | -0.85123 | 0.171657 | 0.604609 |
| m-miR-294_rfam7.0 | -1.00257 | 0.001996 | 0.009863 |
| hmr-miR-27b_rfam7.0 | -1.02676 | 0.001996 | 0.009863 |
| mr-miR-292-3p_rfam7.0 | -1.05051 | 0.001996 | 0.009863 |
| hmr-miR-30c_rfam7.0 | -1.65186 | 0.001996 | 0.009863 |
| mr-miR-34b_rfam7.0 | -2.05151 | 0.001996 | 0.009863 |
| hm-miR-182_rfam7.0 | -2.22217 | 0.001996 | 0.009863 |
| mr-miR-292-5p_rfam7.0 | -3.88404 | 0.001996 | 0.009863 |

**Table S6.**  Comparative marker selection analysis on 4-cell embryos vs. 8-cell embryos. Only SNR scores of > 0.5 or <0.5 are shown.
