# Supplementary material for: microRNA Expression during Trophectoderm Specification
Source: PLoS One. 2009 Jul 3;4(7):e6143. doi: 10.1371/journal.pone.0006143 (PMC2702083; doi:10.1371/journal.pone.0006143)
Supplement: Table S7 — Comparative marker selection analysis on 8-cell vs. morula. Only SNR scores of >0.5 or <0.5 are shown. (0.07 MB DOC) [file pone.0006143.s012.doc]

| Feature | Score | Feature P | FDR(BH) |
| --- | --- | --- | --- |
| mr-miR-34b_rfam7.0 | 5.224381 | 0.197605 | 0.335329 |
| hmr-miR-193a_rfam7.0 | 2.12437 | 0.197605 | 0.335329 |
| hmr-miR-96_rfam7.0 | 1.187657 | 0.197605 | 0.335329 |
| hmr-miR-92_rfam7.0 | 1.00777 | 0.197605 | 0.335329 |
| hm-miR-182_rfam7.0 | 0.858618 | 0.197605 | 0.335329 |
| hmr-miR-30d_rfam7.0 | 0.852126 | 0.197605 | 0.335329 |
| hmr-miR-30c_rfam7.0 | 0.81587 | 0.197605 | 0.335329 |
| hmr-let-7d_rfam7.0 | 0.707107 | 0.826347 | 0.856953 |
| hmr-let-7a_rfam7.0 | 0.707107 | 0.826347 | 0.856953 |
| hmr-miR-204_rfam7.0 | 0.707107 | 0.826347 | 0.856953 |
| hmr-miR-101_rfam7.0 | 0.582837 | 0.826347 | 0.856953 |
| hmr-miR-103_rfam7.0 | 0.571072 | 0.60479 | 0.704645 |
| h-miR-106a_rfam7.0 | 0.563975 | 0.433134 | 0.606387 |
| hmr-miR-33_rfam7.0 | -0.51118 | 0.163673 | 0.335329 |
| hmr-miR-214_rfam7.0 | -0.57735 | 0.001996 | 0.004412 |
| hmr-miR-143_rfam7.0 | -0.57735 | 0.001996 | 0.004412 |
| hmr-miR-29c_rfam7.0 | -0.57735 | 0.001996 | 0.004412 |
| hmr-miR-23a_rfam7.0 | -0.57735 | 0.001996 | 0.004412 |
| hmr-miR-141_rfam7.0 | -0.57735 | 0.001996 | 0.004412 |
| hmr-miR-34c_rfam7.0 | -0.57735 | 0.001996 | 0.004412 |
| hmr-miR-34a_rfam7.0 | -0.57735 | 0.001996 | 0.004412 |
| hm-miR-1_rfam7.0 | -0.57735 | 0.001996 | 0.004412 |
| hmr-miR-196a_rfam7.0 | -0.57735 | 0.213573 | 0.345002 |
| hmr-miR-23b_rfam7.0 | -0.57735 | 0.001996 | 0.004412 |
| mr-miR-290_rfam7.0 | -0.57735 | 0.001996 | 0.004412 |
| hmr-miR-24_rfam7.0 | -0.57735 | 0.001996 | 0.004412 |
| hmr-miR-181a_rfam7.0 | -0.57735 | 0.001996 | 0.004412 |
| mr-miR-351_rfam7.0 | -0.57735 | 0.001996 | 0.004412 |
| hmr-miR-29b_rfam7.0 | -0.57735 | 0.001996 | 0.004412 |
| hmr-miR-449_rfam7.0 | -0.60572 | 0.237525 | 0.369483 |
| m-miR-294_rfam7.0 | -0.60891 | 0.199601 | 0.335329 |
| mr-miR-292-3p_rfam7.0 | -0.61585 | 0.211577 | 0.345002 |
| m-miR-295_rfam7.0 | -0.6319 | 0.001996 | 0.004412 |
| m-miR-199b_rfam7.0 | -0.6388 | 0.199601 | 0.335329 |
| hmr-let-7b_rfam7.0 | -0.67832 | 0.163673 | 0.335329 |
| hmr-miR-323_rfam7.0 | -0.72069 | 0.199601 | 0.335329 |
| hmr-miR-27a_rfam7.0 | -0.84795 | 0.001996 | 0.004412 |
| m-miR-293_rfam7.0 | -0.85776 | 0.001996 | 0.004412 |
| hmr-let-7c_rfam7.0 | -0.90469 | 0.001996 | 0.004412 |
| hsa-miR-503 (j-mir-51) | -1.01273 | 0.001996 | 0.004412 |
| hmr-miR-142-3p_rfam7.0 | -1.0267 | 0.001996 | 0.004412 |
| mr-miR-291-5p_rfam7.0 | -1.03155 | 0.001996 | 0.004412 |
| hmr-miR-195_rfam7.0 | -1.14154 | 0.001996 | 0.004412 |
| mr-miR-291-3p_rfam7.0 | -1.24686 | 0.001996 | 0.004412 |
| hmr-miR-342_rfam7.0 | -1.41819 | 0.001996 | 0.004412 |
| hmr-miR-137_rfam7.0 | -1.42486 | 0.001996 | 0.004412 |
| hmr-miR-30a-5p_rfam7.0 | -1.46135 | 0.001996 | 0.004412 |
| mr-miR-211_rfam7.0 | -1.53617 | 0.001996 | 0.004412 |
| hmr-miR-125a_rfam7.0 | -1.68356 | 0.001996 | 0.004412 |
| hmr-miR-15b_rfam7.0 | -1.70802 | 0.001996 | 0.004412 |
| hmr-miR-99b_rfam7.0 | -1.99357 | 0.001996 | 0.004412 |
| hmr-miR-140*_rfam7.0 | -2.20947 | 0.001996 | 0.004412 |
| mr-miR-292-5p_rfam7.0 | -2.25143 | 0.001996 | 0.004412 |

**Table S7.**  Comparative marker selection analysis on 8–cell vs. morula. Only SNR scores of > 0.5 or <0.5 are shown.
