# Supplementary material for: microRNA Expression during Trophectoderm Specification
Source: PLoS One. 2009 Jul 3;4(7):e6143. doi: 10.1371/journal.pone.0006143 (PMC2702083; doi:10.1371/journal.pone.0006143)
Supplement: Table S8 — Comparative marker selection analysis on morula vs. blastocyst. Only SNR scores of >0.5 or <0.5 are shown. (0.06 MB DOC) [file pone.0006143.s013.doc]

| **Feature** | **Score** | **Feature P** | **FDR(BH)** |
| --- | --- | --- | --- |
| hmr-miR-140*_rfam7.0 | 1.75686 | 0.095808 | 0.309535 |
| mr-miR-211_rfam7.0 | 1.536171 | 0.095808 | 0.309535 |
| hmr-miR-30a-5p_rfam7.0 | 1.130034 | 0.095808 | 0.309535 |
| m-miR-376a_rfam7.0 | 0.845773 | 0.367265 | 0.717605 |
| hmr-miR-101_rfam7.0 | 0.743573 | 0.427146 | 0.717605 |
| hmr-miR-195_rfam7.0 | 0.665042 | 0.427146 | 0.717605 |
| hmr-miR-29b_rfam7.0 | 0.57735 | 1 | 1 |
| hmr-miR-24_rfam7.0 | 0.57735 | 0.996008 | 1 |
| hmr-miR-338_rfam7.0 | 0.57735 | 0.996008 | 1 |
| hmr-miR-143_rfam7.0 | 0.57735 | 0.978044 | 1 |
| hmr-miR-181a_rfam7.0 | 0.549745 | 0.996008 | 1 |
| hmr-miR-23b_rfam7.0 | 0.501532 | 0.996008 | 1 |
| hmr-miR-30d_rfam7.0 | -0.52021 | 0.339321 | 0.712575 |
| hmr-miR-125a_rfam7.0 | -0.5479 | 0.085828 | 0.309535 |
| mr-miR-292-3p_rfam7.0 | -0.55038 | 0.203593 | 0.551671 |
| hmr-miR-18a_rfam7.0 | -0.57328 | 0.107784 | 0.335329 |
| hmr-miR-199a_rfam7.0 | -0.57735 | 0.001996 | 0.008824 |
| mr-miR-34b_rfam7.0 | -0.57735 | 0.001996 | 0.008824 |
| hmr-miR-106b_rfam7.0 | -0.57735 | 0.001996 | 0.008824 |
| hmr-miR-193a_rfam7.0 | -0.57735 | 0.001996 | 0.008824 |
| h-miR-302c_rfam7.0 | -0.57735 | 0.001996 | 0.008824 |
| hmr-miR-96_rfam7.0 | -0.57735 | 0.618762 | 0.951116 |
| hmr-miR-27a_rfam7.0 | -0.60662 | 0.217565 | 0.571108 |
| hmr-miR-99a_rfam7.0 | -0.6176 | 0.131737 | 0.381582 |
| hmr-miR-27b_rfam7.0 | -0.65524 | 0.085828 | 0.309535 |
| hmr-miR-30c_rfam7.0 | -0.70579 | 0.001996 | 0.008824 |
| hmr-let-7a_rfam7.0 | -0.71339 | 0.001996 | 0.008824 |
| hmr-miR-92_rfam7.0 | -0.72706 | 0.085828 | 0.309535 |
| hmr-miR-107_rfam7.0 | -0.86566 | 0.11976 | 0.359281 |
| mr-miR-298_rfam7.0 | -0.89155 | 0.001996 | 0.008824 |
| hmr-let-7d_rfam7.0 | -0.89887 | 0.001996 | 0.008824 |
| hmr-miR-424_rfam7.0 | -0.9726 | 0.203593 | 0.551671 |
| h-miR-10b_rfam7.0 | -1.03337 | 0.001996 | 0.008824 |
| hm-miR-1_rfam7.0 | -1.04546 | 0.085828 | 0.309535 |
| hmr-miR-17-5p_rfam7.0 | -1.06352 | 0.001996 | 0.008824 |
| hmr-miR-16_rfam7.0 | -1.10972 | 0.001996 | 0.008824 |
| h-miR-503_rfam7.0 | -1.11485 | 0.001996 | 0.008824 |
| mr-miR-351_rfam7.0 | -1.27842 | 0.001996 | 0.008824 |
| hmr-miR-34a_rfam7.0 | -1.28006 | 0.001996 | 0.008824 |
| h-miR-106a_rfam7.0 | -1.82784 | 0.001996 | 0.008824 |
| hmr-miR-204_rfam7.0 | -2.08295 | 0.001996 | 0.008824 |
| hmr-miR-15b_rfam7.0 | -4.35351 | 0.001996 | 0.008824 |

**Table S8.**  Comparative marker selection analysis on morula vs. blastocyst. Only SNR scores of > 0.5 or <0.5 are shown.
