# Supplementary material for: microRNA Expression during Trophectoderm Specification
Source: PLoS One. 2009 Jul 3;4(7):e6143. doi: 10.1371/journal.pone.0006143 (PMC2702083; doi:10.1371/journal.pone.0006143)
Supplement: Table S9 — Comparative marker selection analysis on 8-cell vs. blastocyst. Only SNR scores >0.5 or <−0.5 are shown. (0.07 MB DOC) [file pone.0006143.s014.doc]

| **Feature** | **Score** | **Feature P** | **FDR(BH)** |
| --- | --- | --- | --- |
| hmr-miR-30c_rfam7.0 | 0.728075 | 0.197605 | 0.377246 |
| hmr-miR-338_rfam7.0 | 0.707107 | 0.780439 | 0.885904 |
| hmr-miR-101_rfam7.0 | 0.707107 | 0.826347 | 0.90147 |
| m-miR-346_rfam7.0 | 0.614176 | 0.39521 | 0.600063 |
| hmr-miR-92_rfam7.0 | 0.570997 | 0.419162 | 0.607062 |
| m-miR-376a_rfam7.0 | 0.558233 | 0.826347 | 0.90147 |
| hmr-miR-21_rfam7.0 | -0.50857 | 0.373253 | 0.600063 |
| h-miR-215_rfam7.0 | -0.51568 | 0.187625 | 0.366523 |
| hmr-miR-424_rfam7.0 | -0.52946 | 0.163673 | 0.335329 |
| hmr-miR-204_rfam7.0 | -0.55233 | 0.237525 | 0.399042 |
| hmr-miR-107_rfam7.0 | -0.57678 | 0.211577 | 0.383234 |
| hmr-miR-195_rfam7.0 | -0.57735 | 0.001996 | 0.004192 |
| hmr-miR-199a_rfam7.0 | -0.57735 | 0.001996 | 0.004192 |
| hmr-miR-196a_rfam7.0 | -0.57735 | 0.001996 | 0.004192 |
| hmr-miR-181a_rfam7.0 | -0.57735 | 0.001996 | 0.004192 |
| hmr-miR-214_rfam7.0 | -0.57735 | 0.001996 | 0.004192 |
| hmr-let-7c_rfam7.0 | -0.57735 | 0.001996 | 0.004192 |
| hmr-miR-23b_rfam7.0 | -0.57735 | 0.001996 | 0.004192 |
| hmr-miR-34c_rfam7.0 | -0.57735 | 0.001996 | 0.004192 |
| hmr-miR-106b_rfam7.0 | -0.57735 | 0.001996 | 0.004192 |
| h-miR-302c_rfam7.0 | -0.57735 | 0.001996 | 0.004192 |
| hmr-miR-29c_rfam7.0 | -0.57735 | 0.001996 | 0.004192 |
| mr-miR-290_rfam7.0 | -0.60042 | 0.001996 | 0.004192 |
| hmr-miR-142-3p_rfam7.0 | -0.6561 | 0.001996 | 0.004192 |
| hmr-miR-17-5p_rfam7.0 | -0.72811 | 0.223553 | 0.383234 |
| hmr-miR-99a_rfam7.0 | -0.73016 | 0.223553 | 0.383234 |
| h-miR-106a_rfam7.0 | -0.81607 | 0.223553 | 0.383234 |
| m-miR-295_rfam7.0 | -0.81918 | 0.001996 | 0.004192 |
| mr-miR-298_rfam7.0 | -0.84917 | 0.001996 | 0.004192 |
| hmr-miR-18a_rfam7.0 | -0.86213 | 0.223553 | 0.383234 |
| hsa-miR-503 (j-mir-51) | -0.89623 | 0.001996 | 0.004192 |
| hmr-miR-27b_rfam7.0 | -0.90714 | 0.373253 | 0.600063 |
| m-miR-293_rfam7.0 | -0.96924 | 0.001996 | 0.004192 |
| h-miR-10b_rfam7.0 | -1.03337 | 0.001996 | 0.004192 |
| m-miR-294_rfam7.0 | -1.04482 | 0.001996 | 0.004192 |
| hmr-miR-27a_rfam7.0 | -1.05684 | 0.001996 | 0.004192 |
| h-miR-503_rfam7.0 | -1.11485 | 0.001996 | 0.004192 |
| hmr-miR-141_rfam7.0 | -1.11927 | 0.001996 | 0.004192 |
| hmr-miR-23a_rfam7.0 | -1.14243 | 0.001996 | 0.004192 |
| mr-miR-291-5p_rfam7.0 | -1.312 | 0.001996 | 0.004192 |
| hmr-miR-125a_rfam7.0 | -1.50643 | 0.001996 | 0.004192 |
| mr-miR-292-3p_rfam7.0 | -1.60919 | 0.001996 | 0.004192 |
| hmr-miR-16_rfam7.0 | -1.70324 | 0.001996 | 0.004192 |
| mr-miR-292-5p_rfam7.0 | -2.24303 | 0.001996 | 0.004192 |
| mr-miR-351_rfam7.0 | -3.22983 | 0.001996 | 0.004192 |
| mr-miR-291-3p_rfam7.0 | -3.26334 | 0.001996 | 0.004192 |
| hmr-miR-34a_rfam7.0 | -6.01902 | 0.001996 | 0.004192 |
| hmr-miR-15b_rfam7.0 | -6.30706 | 0.001996 | 0.004192 |
| hm-miR-1_rfam7.0 | -6.32779 | 0.001996 | 0.004192 |
| hmr-miR-137_rfam7.0 | -6.8238 | 0.001996 | 0.004192 |
| hmr-miR-99b_rfam7.0 | -7.13823 | 0.001996 | 0.004192 |

**Table S9.** Comparative marker selection analysis on 8-cell vs. blastocyst. Only SNR scores > 0.5 or < -0.5 are shown.
