# Supplementary material for: microRNA Expression during Trophectoderm Specification
Source: PLoS One. 2009 Jul 3;4(7):e6143. doi: 10.1371/journal.pone.0006143 (PMC2702083; doi:10.1371/journal.pone.0006143)
Supplement: Table S10 — Predicted target genes using (miRBASE)for candidate miRNAs involved in trophectoderm specification were classified by gene ontology. Pathways with p<0.05 are shown. (0.03 MB DOC) [file pone.0006143.s015.doc]

**Pathways** # # **expected** **+/-** P value

Unclassified 26616 5994 6192.93 - 1.80E-11

Inflammation (chemokine/cytokine signaling) 337 134 78.41 + 9.72E-07

Integrin signalling pathway 263 110 61.19 + 1.77E-06

Interleukin signaling pathway 169 72 39.32 + 2.92E-04

PDGF signaling pathway 186 77 43.28 + 3.66E-04

Angiogenesis 258 97 60.03 + 1.06E-03

p53 pathway feedback loops 2 62 33 14.43 + 3.10E-03

FGF signaling pathway 143 59 33.27 + 5.65E-03

Endothelin signaling pathway 97 43 22.57 + 1.34E-02

2-arachidonoylglycerol biosynthesis 8 9 1.86 + 2.33E-02

Coenzyme A biosynthesis 8 9 1.86 + 2.33E-02

Dopamine receptor mediated signaling pathway 83 37 19.31 + 3.62E-02

B cell activation 96 41 22.34 + 4.10E-02

**Table S10.** Predicted target genes using (miRBASE)for candidate miRNAs involved in trophectoderm specification were classified by gene ontology. Pathways with p < 0.05 are shown.
